# Analytical Chemistry and Material Purity in the Semiconductor Industry

**DOI:** 10.6028/jres.093.091

**Published:** 1988-06-01

**Authors:** Purneshwar Seegopaul

**Affiliations:** Materials Research Corporation, Orangeburg, NY 10962

Analytical chemistry has evolved from a “hodge-podge” of empirical ideas into a highly visible, diverse and ubiquitous science. Perhaps the two most important industries that place significant demands on analytical chemistry are the biomedical and semiconductor fields. One can envisage a symbiotic relationship between analytical chemistry and the semiconductor industry. While improved analytical methods and instrumentation are constantly needed to support technological progress, it is technology development that provides the impetus and tools for enhanced instruments and thereby, analytical methodology.

The electronic revolution has resulted in significant miniaturization of devices on integrated circuits with complex multi-level structures. A typical VLSI circuit may contain up to a million devices! Obviously, this degree of complexity raises concern of yield, quality, and reliability. Materials play a critical role in these issues with the type and purity of the material directly influencing the profitability and very survival of semiconductor industries. For example, capacitance-voltage shifts caused by mobile ion impurities and ionization effects resulting from alpha-particles destroy the integrity of the devices. Table 1 shows the diversity of materials and their applications.

The enormous significance of material purity, however, is unfortunately clouded with the current practice of defining purity. At best, the purity phenomenon exists in a very arbitrary manner with its use subject to a myriad of interpretations. Purity of material in the semiconductor industry generally refers to the extent of the absence of impurities in the material. This then introduces the concept of total purity.

The ambiguity in purity data stems from the arbitrary and injudicious use of nine’s without reference to acceptable and defined specifications. This method yields total purity by simply cumulative total impurity level from 100% to give the data in nine’s. This is shown in table 2. It all seems too easy and simple! What does it mean? Was a six nine’s (99.9999) pure material exhaustively analyzed and found only to contain I ppm total impurity out of all possible contaminants?

The system of defining, describing, and interpretating purity data needs to be modified. Such changes should be brought about by joint efforts of National Organizations, such as, NBS and ASTM in conjunction with material suppliers and users. Material purity, however, cannot be considered as an isolated issue. It is intricately linked to all the processes and techniques utilized to generate and guarantee the purity data. Table 3 lists some of the areas that are related to the whole concept of purity.

Specifications that define purity are generally built with the manufacturing capability in conjunction with the material application. These specifications must be realistic and clearly defined with a critical list of parameters for evaluation; for example, a critical elemental list. The use of nine’s may then become more practical.

Perhaps the most important area in this purity debate is the analytical process. Poor analytical practice and techniques generate useless data that distort the information. The analytical chemist is fortunate to have an impressive repertoire of modern techniques to discharge these responsibilities. Such techniques range from the simple potentiometric ion selective electrodes to the sophisticated glow discharge mass spectrometer. It is interesting to note that the choice of technique can have a direct influence on the purity in terms of the sensitivity and detection limits of the methods.

The interpretation and communication of the analytical data introduce the “numbers game.” Data manipulation cannot be done without clearly defined conditions. Significant figures, use of absolute or rounded-off numbers, allowable deviations from the specified limits, etc., influence the final result and therefore, the purity. Confusion also arises from use of analytical parameters that are not in accordance with regulations, if any. For example, detection limits are being used with two and three standard deviations. Certification of the analytical data must indicate the use of actual results as opposed to typical values. The use of actual analysis data is more practical in terms of the risks involved and users of high purity materials must be aware of the significant difference in these numbers in their material and supplier evaluation.

The other critical consideration in the purity debate is the subject of reliability. Basically, this involves the need for comprehensive analytical quality control. Table 4 describes some of these parameters which include both internal laboratory and external control. Constant monitoring of accuracy and precision of procedures and data in conjunction with other aspects of the analytical process should be established as standard operating procedures. The use of reference samples and duplicates in analysis are absolutely critical for reliability which in turn produces real and useful information on material purity. A recent trend in the industry is the growing need for statistical process control (SPC). Obviously, SPC cannot be effectively utilized without accurate and precise data. Control charts are now necessary for certification of material purity! Material purity data is now being integrated with the actual process stability to monitor consistency.

The criticality of material purity, therefore, demands that the concept be clearly defined and specified for effective use and correlation of purity data. This discussion highlights the issues that need to be resolved and focused on the role of the analytical chemistry in the process.

## Figures and Tables

**Table 1:** Materials and their applications

	
Thermal print heads		Optical disks	
Ta_2_O_5_	Au	Te	CaF_2_
SiC	Al	Bi	
SiO_2_	Cr	Al	
TaN		Rh	
		And alloys of above	
	

	
Thin film heads		Hard disks—Winchester type	
Ni-Fe	Al_2_O_3_	Ni-Fe	S_i_O_2_
Fe-Si-Al	Au	Co-Ni	Cr
SiO_2_		Co-Cr	Ni-V
Al		C	
		Co-Ni-Cr	
	

	
Integrated circuits		Optical magnetic disks
Al(Al-Si. Al-Cu, Al-Si-Cu)		Gd-Co Tb-Fe
Si	Mo & MoSi_2_	Al
W-Ti	Ta & TaSi_2_	SiO_2_
SiO_2_	W & WSi_2_	Th-Co-Fe
Au	Ti & TiSi_2_	
Pt	Doped Si	And other alloys
	

	
Liquid crystal displays		Thin film hybrids	
ITO	SiO_2_	Cr	Au
ATO	Al_2_O_2_	Ni-V	TaN
Doped ZnS	Al	W-Ti	Ta
In-Sn Metal			
	

	
Resistors		Image sensors	
Hybrids	Monolithic		
	
Ni-Cr	Al-Ta	ITO	Cr
Cr-Si		In-Sn	Al
Cr/Cr_2_0_3_	Ni-Cr-Si	SiO_2_	
Cr-SiO_2_	Ni-Cr-Al		
	

**Table 2:** Classification of purity by use of nine’s

Three nine’s	--	3N	--	99.9%	--	1000 ppm impurities
Four nine’s	--	4N	--	99.99%	--	100 ppm impurities
Five nine’s	--	5N	--	99.999%	--	10 ppm impurities
Six nine’s	--	6N	--	99.9999%	--	1 ppm impurities

**Table 3:** Considerations in the purity debate

• Semantics	—	Meaning Comprehension/interpretation
• Specifications	—	Reference to purity Needs and material application
• Measurement process	—	Techniques Related functions
• Data reduction	—	“Numbers” game
• Data communication	—	Analytical terminology Certification of purity actual vs typical Correlation of purity data
• Reliability	—	Laboratory QC SPC Process stability and purity

**Table 4:** Reliability of the analytical process

Analytical quality control Sampling * Valid methods statistics Sample/data management * Accountability * Traceability * Automation—LIMS * Terminology and interpretation of data Internal quality control * Procedures * Accuracy * Precision * Facilities/personnel External quality control * Round Robin Studies * Accreditation Statistical process control Process stability Purity vs consistency

